# Stimulus-dependent differences in signalling regulate epithelial-mesenchymal plasticity and change the effects of drugs in breast cancer cell lines

**DOI:** 10.1186/s12964-015-0106-x

**Published:** 2015-05-15

**Authors:** Joseph Cursons, Karl-Johan Leuchowius, Mark Waltham, Eva Tomaskovic-Crook, Momeneh Foroutan, Cameron P Bracken, Andrew Redfern, Edmund J Crampin, Ian Street, Melissa J Davis, Erik W Thompson

**Affiliations:** Systems Biology Laboratory, Melbourne School of Engineering, University of Melbourne, Building 193, Parkville, VIC 3010 Australia; ARC Centre of Excellence in Convergent Bio-Nano Science and Technology, Melbourne School of Engineering, University of Melbourne, Parkville, VIC 3010 Australia; The Walter and Eliza Hall Institute of Medical Research, 1G Royal Parade, Parkville, VIC 3052 Australia; Department of Medical Biology, The University of Melbourne, Parkville, VIC 3010 Australia; St. Vincent’s Institute, Melbourne, VIC Australia; Centre for Cancer Biology, SA Pathology and University of South Australia, Adelaide, SA 5000 Australia; Discipline of Medicine, University of Adelaide, Adelaide, SA 5005 Australia; Royal Perth Hospital, Perth, Australia; School of Mathematics and Statistics, Faculty of Science, University of Melbourne, Parkville, VIC 3010 Australia; School of Medicine, Faculty of Medicine Dentistry and Health Sciences, University of Melbourne, Parkville, VIC 3010 Australia; Institute of Health and Biomedical Innovation and School of Biomedical Sciences, Queensland Institute of Technology, Brisbane, Australia; University of Melbourne Department of Surgery, St. Vincent’s Hospital, Melbourne, Australia

**Keywords:** Epithelial mesenchymal plasticity, EMT, Metastasis, Breast cancer, EGF, Hypoxia, MEK, AKT, MDA-MB-468

## Abstract

**Introduction:**

The normal process of epithelial mesenchymal transition (EMT) is subverted by carcinoma cells to facilitate metastatic spread. Cancer cells rarely undergo a full conversion to the mesenchymal phenotype, and instead adopt positions along the epithelial-mesenchymal axis, a propensity we refer to as *epithelial mesenchymal plasticity* (EMP). EMP is associated with increased risk of metastasis in breast cancer and consequent poor prognosis. Drivers towards the mesenchymal state in malignant cells include growth factor stimulation or exposure to hypoxic conditions.

**Methods:**

We have examined EMP in two cell line models of breast cancer: the PMC42 system (PMC42-ET and PMC42-LA sublines) and MDA-MB-468 cells. Transition to a mesenchymal phenotype was induced across all three cell lines using epidermal growth factor (EGF) stimulation, and in MDA-MB-468 cells by hypoxia. We used RNA sequencing to identify gene expression changes that occur as cells transition to a more-mesenchymal phenotype, and identified the cell signalling pathways regulated across these experimental systems. We then used inhibitors to modulate signalling through these pathways, verifying the conclusions of our transcriptomic analysis.

**Results:**

We found that EGF and hypoxia both drive MDA-MB-468 cells to phenotypically similar mesenchymal states. Comparing the transcriptional response to EGF and hypoxia, we have identified differences in the cellular signalling pathways that mediate, and are influenced by, EMT. Significant differences were observed for a number of important cellular signalling components previously implicated in EMT, such as HBEGF and VEGFA.

We have shown that EGF- and hypoxia-induced transitions respond differently to treatment with chemical inhibitors (presented individually and in combinations) in these breast cancer cells. Unexpectedly, MDA-MB-468 cells grown under hypoxic growth conditions became even more mesenchymal following exposure to certain kinase inhibitors that prevent growth-factor induced EMT, including the mTOR inhibitor everolimus and the AKT1/2/3 inhibitor AZD5363.

**Conclusions:**

While resulting in a common phenotype, EGF and hypoxia induced subtly different signalling systems in breast cancer cells. Our findings have important implications for the use of kinase inhibitor-based therapeutic interventions in breast cancers, where these heterogeneous signalling landscapes will influence the therapeutic response.

**Electronic supplementary material:**

The online version of this article (doi:10.1186/s12964-015-0106-x) contains supplementary material, which is available to authorized users.

## Introduction

Epithelial mesenchymal transition (EMT) is the directional process where sessile, polarised epithelial cells alter the expression of key adhesion and regulatory molecules and gain the ability to survive and migrate as single cells. EMT is a normal process that occurs early in development to generate the primary mesenchyme, and later in the ectoderm to form muscle, bone, nerve and connective tissues [1]. In development, EMT is transient, and the phenotypic shift is followed by the reverse transition (MET) at the target site [1,2]. Metastasis is now recognized to have many elements in common with developmental EMT, such as single cell dispersal, increased migratory and invasive potential, and gene expression changes [2-5]. When these transitions occur in cancer, however, a hybrid/metastable phenotype is reached after the carcinoma undergoes a subtle EMT, rather than full mesenchymal conversion [6-8]. We use the term *epithelial mesenchymal plasticity* (EMP) for phenotypic flux of cancer cells along the EMT-MET axis, as they shift between organized, polarized, sessile epithelial cells and more individual and motile mesenchymal cells, facilitating metastatic spread [5,6,9,10].

Specific support for the importance of EMP in breast cancer (BrCa) pathogenesis comes from the observations that BrCa stem cells (BCSC) exhibit a mesenchymal phenotype [5,11-13]. BCSC exhibit dramatically enhanced malignant/metastatic properties compared to their non-BCSC counterparts, and can regenerate a heterogeneous tumour cell population [14,15]. They overexpress CD44, have low expression of the luminal marker CD24 (CD44^hi^CD24^lo/-^), and have a transcription profile resembling EMT-transformed cells [13,16]. Basal subtypes of BrCa, which have a poor prognosis, exhibit increased EMT marker expression [17]. The links between EMT, BCSC, and basal breast cancer therefore place EMP at the mechanistic core of the most malignant cells found in clinical BrCa. Further to this, in breast cancer patients EMT correlates with adverse prognosis. An EMT signature was found to predict delayed relapse using available on-line data in 4767 breast cancer tumour samples [18]. In multiple studies, poor patient outcomes have been shown to be correlated with the altered expression of various protein markers of EMT development, including increased vimentin [19], loss of certain epithelial cytokeratins [20], loss of E-cadherin and gain of N-cadherin [21]. Additionally, EMT can be induced in patient breast cancers in response to standard chemotherapies [22] and hormonal therapies [23], suggesting a potential role for EMT in treatment resistance.

EMT is known to be controlled by a set of transcription factors including SNAI1/2, ZEB1/2, and other basic helix-loop-helix factors, which coordinate programs of gene expression during EMT (reviewed in [24,25]). Demonstrating the importance of these pathways in treatment outcome, work by a number of groups has shown that over-expression of SNAI1/2, or TWIST1 in breast cancer cells results in both EMT and chemoresistance [26-28]. The activity of these transcription factors is controlled through a number of signalling pathways that sense changes to the cellular environment and initiate cascades of signalling that result in transcriptional activation or repression. The stimuli that trigger these regulators to induce EMT vary. Signalling through EGFRs is a well-established driver of breast cancer progression [29,30], and EGF is also known to stimulate EMT in some cells [3,31-35]. Hypoxia has been shown to induce EMT through HIF1a activation of TWIST in a variety of cell lines [36,37], and through SNAI1 in hepatocellular carcinoma [38]. Furthermore, dysregulated signalling through pathways such p38 MAPK [39] and PI3K-Akt [28,40] has been implicated in EMP regulation. Because such signalling pathways are often druggable, they represent important targets for novel therapeutics. For example, considerable interest has been generated in recent years by classes of kinase inhibitors that are able to modulate cellular signalling and interrupt oncogenic signalling. This motivates the question: *if multiple stimuli are able to trigger the more aggressive mesenchymal phenotype in cancer cells, do the responses to these stimuli converge upon common signalling elements, or do they achieve similar phenotypic outcomes through distinctly different molecular pathways?* The answer to this question has clear implications for the design of molecular targeted therapies, as well as for managing the fundamental heterogeneity of breast cancer.

We have employed two human BrCa cell line models of stable (PMC42) and dynamically induced (MDA MB 468) EMP. PMC42-LA is an epithelial subline derived from the vimentin^+^, E-Cadherin^−^ parental PMC42-ET cells [41,42]. PMC42-LA cells demonstrate heterogeneity where approximately 90% of the cells are E-cadherin^+^ while the remaining 10% lack E-cadherin and are vimentin^+^. PMC42-LA cells undergo a marked EGF-induced EMT [3,42,43]. PMC42-ET cells also undergo EGF-driven EMT, however the change in expression of mesenchymal markers is reduced due to higher basal expression of markers such as vimentin [3,42]. When compared to other BrCa cell lines [44], both PMC42-ET cells and the LA subline exhibit a “Basal B” (mesenchymal) transcriptome, clustering together despite their EMP differences, and irrespective of EGF treatment (E Tomaskovic-Crook & T Blick, unpublished observation). MDA-MB-468 cells have a “Basal A” transcriptome [44] indicating mixed luminal/basal attributes, and although predominantly epithelial and E-cadherin^+^, they lack α-catenin and tight junction protein 1 (TJP1, or ZO-1). About 5% of these cells are vimentin^+^ and they display intermediate invasiveness [45]. MDA-MB-468 cells also exhibit a marked EGF-regulated EMT, as well as hypoxia-driven EMT (2% O_2_) [42,46,47]. MDA-MB-468 xenografts exhibit distinct zones of mesenchymal transition, one at the stromal periphery and the other at the interface with the central necrosis common in this xenograft model [48,49].

These models provided an opportunity to investigate both differential lineage-specific cellular responses to the same EMT-promoting stimulus as well as differential responses to varied EMT promoting stimuli in the same cell line. By observing the transcriptional changes in PMC42 cells and in MDA-MB-468 cells under different stimuli, we were able to identify patterns of disruption that are distinct to each stimulus as well as common to all. These observations have clear implications for the therapeutic benefit of pharmaceutical manipulation of these pathways during cancer progression. We tested this notion using drugs targeting these key pathways and demonstrated clear differences in the extent to which different drugs are able to block mesenchymal transition induced by different triggers. The divergence of signalling for EMP regulation between EGF and hypoxia that we characterise here is of therapeutic importance, particularly bearing in mind associations in breast cancer patients between EMT, poor prognosis and treatment resistance.

## Materials and methods

### Cell Lines

PMC42-ET (ET) cells were derived from a breast cancer pleural effusion by Dr. Robert Whitehead, Ludwig Institute for Cancer Research, Melbourne, Australia, with appropriate institutional ethics clearance (Institutional Review Board of the Peter MacCallum Hospital, Melbourne) and informed consent of the patient. The PMC42-LA (LA) subline was derived further from the parental PMC42- ET cells by Dr. Leigh Ackland, Deakin University, Melbourne, Australia [50-53]. MDA-MB-468 cells originally from the ATCC were transferred from the Lombardi Cancer Center, Washington, DC, USA.

### Immunofluorescence staining of cells

PMC42-LA and PMC42-ET cells were cultured in RPMI 1640 medium with 10% FBS. MDA-MB-468 cells were cultured in DMEM with 10% FBS. The cell lines had all tested negative for mycoplasma infection. Cells were seeded in 384 well flat bottom microtiter plates (#3712, Corning Life Sciences) and allowed to adhere overnight at 37°C/5% CO_2_. The next day, where indicated in the text, human EGF (#8916, Cell Signaling Technology) was added to the cells at a final concentration of 10 ng/mL. After 72 h the cells were fixed with 3.7% formaldehyde in PBS for 10 minutes, then washed with Tris-buffered saline (TBS). The cells were incubated with a blocking solution of 0.3% Triton X-100 (Sigma-Aldrich) and 5% sterile filtered goat serum (Sigma-Aldrich). Next, cells were incubated overnight at 4°C with mouse anti-vimentin antibody (V6630, Sigma-Aldrich), diluted 1:1600 in TBS with 1% BSA (Sigma-Aldrich) and 0.05% Tween-20 (Sigma-Aldrich). Where indicated, a rabbit anti-phospho-ERK1/2 antibody (#4370, Cell Signaling Technology) was also included at a dilution of 1:200. The cells were washed three times for 5 min with washing buffer (1xTBS with 0.05% Tween-20) then stained with an Alexa-594 conjugated goat-anti mouse antibody (115-585-146, Jackson ImmunoResearch) diluted 1:200 in TBS with 1% BSA and 0.05% Tween-20. To stain the nuclei of the cells, Hoechst 33342 (Sigma-Aldrich) was included at a concentration of 3 μg.mL^−1^. When the pERK1/2 primary antibody was included, an Alexa-488 conjugated goat-anti rabbit antibody (#4412, Cell Signaling Technology) diluted 1:1000 was included in the secondary antibody mix. The cells were stained for 2 hours then washed 3 × 5 minutes with washing buffer.

The plates were imaged on a Perkin Elmer Operetta using a 10X/0.4 NA air objective and the appropriate excitation and emission filters. Excitation time and intensity were set to avoid overexposed pixels in the measured images. Acquired images were analysed using Perkin Elmer Harmony 3.5 software. Cell nuclei were segmented using the Hoechst images, and the nuclear masks were expanded to cover the cytoplasm of the cells. The mean and total fluorescence intensities of vimentin and phospho-ERK were measured within the masked areas, using image data from the corresponding fluorescently labelled secondary antibodies. Cells were then classified as vimentin^+^or vimentin^−^ using a decision tree classifier. The decision tree classifier used the mean and total vimentin intensities of the cells to determine thresholds that maximised the separation of the cell populations between unstimulated wells, and stimulated-cell control wells (16 positive and 16 negative control wells were included for each plate). The number of cells imaged, the percent vimentin^+^ cells and the average phospho-ERK intensity per cell were then calculated for each well. The percent inhibition was calculated as 100*(1-(x-mean(*x*_*pos*_))/(mean(*x*_*neg*_) – mean(*x*_*pos*_)), where *x* indicates the measured variable and mean(*x*) indicates the mean of the measured variable x among the positive or negative control wells. The dose response curves of the inhibitors tested for the different cell lines and measured variables were fitted and plotted in GraphPad Prism 6. Cooperativity between inhibitors tested in combinations were calculated according to the Median effects method [54] using the CalcuSyn software (Biosoft).

### Kinase inhibitor treatment of cells

Kinase inhibitors were purchased from Selleck Chemicals and were diluted in DMSO then added to the cells at the concentrations indicated within each figure. For determining IC50 values across the range of kinase inhibitors, compounds were serially diluted 1:2 to produce eleven concentrations, with the highest concentration being 10 μM. The final concentration of DMSO in the wells was 0.5%. Positive and negative control wells were included for each plate where the cells were treated with 1 uM of Erlotinib (Selleck Chemicals) or 0.5% DMSO, respectively. For the hypoxia-treated cells, cells grown under normoxic conditions were used as negative controls. The cells were grown in a humidified atmosphere at 37°C/5% CO_2_ for 72 h. Hypoxia-treated cells were grown in a hypoxic chamber at 37°C/5% CO_2_ with 1% O_2_ for 72 h.

### Transcript abundance data

A detailed description of the transcriptome analysis is given elsewhere (Tomaskovic-Crook E, Philip G, Blick T, van Denderen BJW, Haviv I, Thompson EW: RNA Sequencing of Induced Epithelial-Mesenchymal Transition in Breast Cancer Cell Lines, *in preparation*). Briefly, an epithelial-to-mesenchymal transition was induced for PMC42-LA and -ET cell lines through EGF stimulation, and in MDA-MB-468 cells through EGF stimulation or growth under hypoxic conditions. All treatments were applied for 72 h, then RNA was collected from unstimulated and stimulated cells, and mRNA abundances were measured using RNASeq.

Transcript abundance data were compared between unstimulated and stimulated conditions described above: PMC42-ET −/+EGF, PMC42-LA −/+EGF, MDA-MB-468 −/+EGF, MDA-MB-468 −/+HPX. Three ‘inter-model’ comparisons were also performed, between: PMC-42-ET *versus* PMC-42-LA with and without EGF, and MDA-MB-468 HPX *versus* EGF stimulation. These comparisons are arranged such that they are consistent with a ‘general EMT’ process, as classified by vimentin (VIM) up. Sequence alignment was performed using TopHat and differential analysis was performed using CuffDiff. Transcript abundances and test statistics were imported into the MATLAB scripting language (R2012b) for subsequent analysis and to generate heat map plots.

### Pathway analysis

Kyoto Encyclopedia for Genes and Genomes (KEGG) maps were downloaded and gene lists were extracted from associated KGML files. For the over-representation analysis (ORA), all maps annotated as signalling pathways or systems were included. The expected and observed numbers of differentially expressed mRNA transcripts (*q-value* < 0.01) were compared using a *χ*^2^-test within each condition comparison as outlined above. A Bonferoni correction was applied, such that the estimated *p*-values were multiplied by 22 (the number of signalling pathways tested).

### Druggable target and protein-protein interaction networks

Protein interaction networks provide a wider coverage of molecular interactions than are captured by canonical signalling pathways. The Bionet R package [55] was used to compute the top-scoring network in each experiment. First we downloaded the set of human protein interactions provided by the PINA2 website [56] and extracted the network corresponding to proteins encoded by transcripts measured in our MDA-MB-468 experiments (transcripts for which we have no data were excluded from the network). Both the network and the p values from the differential expression analysis were passed to the Bionet package, and we used the *runfastheinz* function to generate the top scoring network for EGF and hypoxia-induced EMP. Networks were exported in the *simple interaction format* (.sif) for analysis in Cytoscape 3.1 [57].

The two resulting networks, based on differential expression induced by EGF and Hypoxia, were merged using the Advanced Network Merge functions in Cytoscape, which we also used to calculate node degree. Data on drugs or compounds and their known targets (and where available their mechanism of action) was downloaded from the Drugbank Database (v3.0) [58] and mapped onto our network using the gene name attributes associated with both drug targets and proteins. These data were used to identify druggable proteins within each network. Nodes were then ranked by their degree within this resulting network and druggable nodes selected for further analysis.

## Results

### Induced epithelial-to-mesenchymal transitions promote a similar cellular phenotype, but act through cell-line and stimulus-specific signalling mechanisms

**Figure 1:**
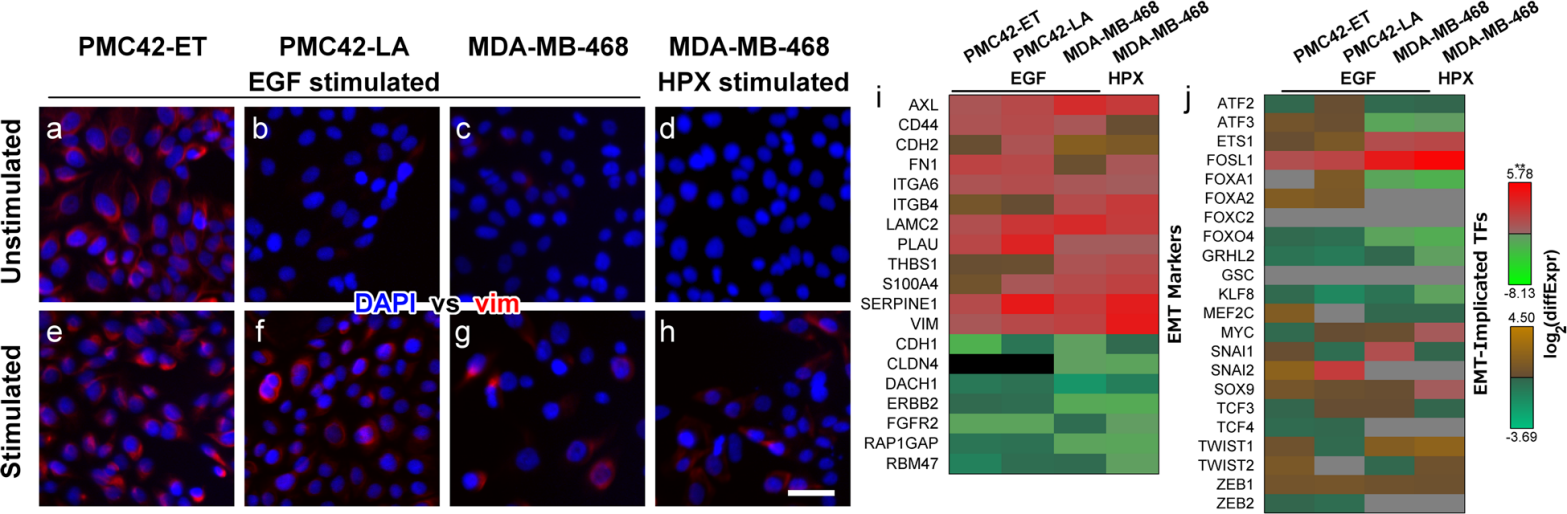
Stimulation of PMC42-ET and PMC42-LA cells with EGF, or stimulation of MDA-MB-468 cells with EGF or growth under hypoxic conditions (HPX) promotes a mesenchymal phenotype. **(a-h)** Fluorescence images of stimulated and unstimulated cells labelled with DAPI (*blue*) and anti-vimentin (*red*). Scale bar represents 10 μm. Changes in mRNA transcript abundance between stimulated and unstimulated cells for **(i)** EMT markers and **(j)** EMT-implicated transcription factors. Note the use of alternative colour-bars to indicate statistically significant (**; *q*-value < 0.05; *red-green*) and non-significant (*brown/orange*-*teal*) changes in abundance. Grey squares indicate mRNA transcripts that were not reliably detected – normalised count data are shown in Additional file 2: Figure S1.

The stimulation of PMC42-LA and MDA-MB-468 cells with EGF, or growth of MDA-MB-468 cells under hypoxic conditions (HPX) each promoted EMT as indicated by an increased proportion of vimentin + cells (*red fluorescence*; Figure 1b & f, c & g, d & h). Unstimulated PMC42-ET cells express vimentin (Figure 1a), thus increases in the number of vimentin^+^ cells with EGF stimulation (Figure 1e) are relatively small, consistent with our previous reports on EMT within this cellular system [40,41].

Examining changes in transcript abundance that occurred with the phenotypic EMT (Figure 1a-h) consistent differences were observed for several transcripts that contribute to the canonical mesenchymal phenotype (Figure 1 & j). Transcripts for VIM were significantly increased in all models of induced EMT, including EGF-stimulated PMC42-ET cells, while several other regulatory/signalling components implicated in EMT [13,59-61] (*further details in Additional file *1*: Table S1*) showed consistent changes with various degrees of significance (Figure 1i). A number of transcripts encoding cellular signalling components implicated in EMT also showed large changes between some of the experimental models as detailed in the sections below.

The mRNAs of transcription factors (TFs) implicated in EMT was also examined and only FOSL1 (also known as FRA1) showed significant increases in transcript abundance across all models of induced EMT (Figure 1j). TFs known to play a role in EMT including ETS1, SOX9 and ZEB1 showed consistent increases in transcript abundance, while FOXO4, KLF8 and the epithelial TF GRHL2 were consistently reduced; however, not all of these changes were statistically significant (Figure 1j). Conversely, several well-studied TFs which drive EMT, such as SNAI1 and TWIST1, showed vastly different expression profiles between differing cell lines and differing stimuli, while ZEB2 and SNAI2 were not reliably detected within the MDA-MB-468 cells, nor were FOXC2 and GSC detected across all cell lines tested (Figure 1j). Furthermore, normalised count data suggest that the mammary stem cell TF SOX9 was much more abundantly expressed in the MDA-MB-468 cells, while TWIST1 and ZEB1 had much higher transcript counts in the PMC42 sublines (Additional file 2: Figure S1).

These results indicate that a phenotypically-similar EMT process was induced across these different cell lines and stimuli, with consistent changes in the transcripts which mediate these canonical changes, such as VIM, CD44, CDH1 and CDH2. However, variation in the differential abundance patterns observed for specific EMT-implicated TFs suggests that these similar phenotypic behaviours are associated with different regulatory mechanisms.

### Pathway analysis highlights alternative signalling mechanisms which contribute to EMT

To identify signalling pathways likely to be affected by the transcriptional changes associated with each model of induced EMT, we first assessed the mRNA transcripts that responded within each model and then mapped these to KEGG pathways. EGF stimulation of PMC42-ET cells led to significant (*q*-value < 0.05) changes in abundance for 238 transcripts (Table 1). This was the lowest number across all of our *in vitro* models of EMT, consistent with PMC42-ET cells being relatively mesenchymal in the unstimulated state (Figure 1**a**). The EGF- and HPX-stimulated MDA-MB-468 cells had significant changes in abundance for 3155 and 3716 transcripts, respectively, indicating a much greater response than the EGF- stimulated PMC42-ET or PMC42-LA cells (Table 1). The number of transcripts with differential abundance for the stimulated MDA-MB-468 cells was of a similar magnitude to the inter-model comparisons between PMC42-ET and -LA sublines in the presence or absence of EGF (3261 and 2938, respectively; Table 1). These inter-model comparisons also show that the number of transcripts with a significantly different abundance between the PMC42-ET and -LA sublines decreased with EGF stimulation, suggesting a potential convergence of phenotypes.

**Table 1:** **Different signalling pathways are dysregulated between the models of** ***in vitro*** **induced EMT**

***differential analysis between:***	**Cell Lines**	**PMC42-ET**	**PMC42-LA**	**MDA-MB-468**	**MDA-MB-468**	**PMC42-ET vs. PMC42-LA**	**PMC42-ET vs. PMC42-LA**	**MDA-MB-468**
	**Conditions**	**+EGF vs. Unstim.**	**+EGF vs. Unstim.**	**+EGF vs. Unstim.**	**+HPX vs. Unstim.**	**Unstim.**	**+EGF**	**+HPX vs +EGF**
	*number of differentially expressed transcripts*	238	591	3155	3716	3261	2938	1626
KEGG signalling pathway/system:	PI3K-Akt	0.015	**0.000**	0.030	0.005	0.153	0.559	0.943
	HIF-1	0.952	0.244	**0.002**	**0.002**	0.982	0.732	**0.000**
	Rap1	0.562	0.014	**0.000**	**0.000**	0.175	0.075	0.742
	Hippo	0.173	**0.000**	0.022	0.085	0.004	0.297	0.996
	Wnt	0.015	0.371	0.038	0.071	**0.002**	0.455	0.534
	MAPK	0.214	0.044	0.312	0.680	0.360	0.634	0.768
	Hedgehog	0.977	**0.000**	0.999	0.939	0.217	0.093	0.993
	TGF-beta	0.029	**0.000**	0.215	0.328	0.053	0.632	0.694
	Ras	1.000	0.169	0.259	0.022	0.120	0.065	0.783
	Phosphatidyl-inositol	0.796	0.833	0.238	0.049	0.753	0.923	0.893
	cAMP	0.992	0.639	0.242	0.017	0.983	0.756	0.201

The first row shows the number of mRNA transcripts with a significant (q-value < 0.05) difference in abundance between the specified cell lines or conditions. Subsequent rows show the estimated *p*-value for enrichment of elements within KEGG pathways without correction for multiple hypothesis testing. KEGG maps related to signal transduction with a significant (*p*-value < 0.05) enrichment in at least one comparison are shown (for a complete list please refer to Additional file 8: Table S2). Values in bold are statistically-significant following a Bonferroni correction for multiple hypothesis testing (adjusted *p*-value < 0.05; n = 22 ‘signalling pathway’ KEGG maps), *p*-value entries greater than 0.10 are in grey.

Next we examined the putative signalling effects of these altered transcript abundances, performing an over-representation analysis to identify intracellular signalling pathways that may have been perturbed (*p*-value < 0.05) by concerted changes to numerous signalling components during induced EMT. Eleven signalling pathways showed some evidence of dysregulation (*p* < 0.05) within at least one model of induced EMT (Table 1). The PI3K-Akt signalling pathway was the only pathway that showed perturbation of components across all models of induced EMT (Table 1); however, after further correcting for multiple hypothesis testing, the EGF-stimulated PMC42-LA cells remained as the only experimental system showing significant transcriptional dysregulation of PI3K-Akt signalling components. The results shown in Table 1 support the observation that although a phenotypically-similar EMT is induced (Figure 1e-h & 1i), as extensively characterised in previous reports by us and others [3,41-43,46-48], there are differences in the molecular mechanisms that drive these phenotypic changes (Figure 1j).

Both the HIF-1 signaling pathway and Rap1 signaling pathway showed very strong transcriptional perturbations within EGF or HPX-stimulated MDA-MB-468 cells, and there was also evidence of HIF-1 signaling pathway dysregulation between EGF and HPX-stimulated MDA-MB-468 cells (Table 1). Strong dysregulation of Hippo, Hedgehog and TGF-beta signalling components was observed with EGF induced EMT within the PMC42-LA cells, and in the absence of EGF, components of the Wnt signalling pathway showed strong differences in transcript abundance between the PMC42-ET and PMC42-LA sublines (Table 1).

**Figure 2:**
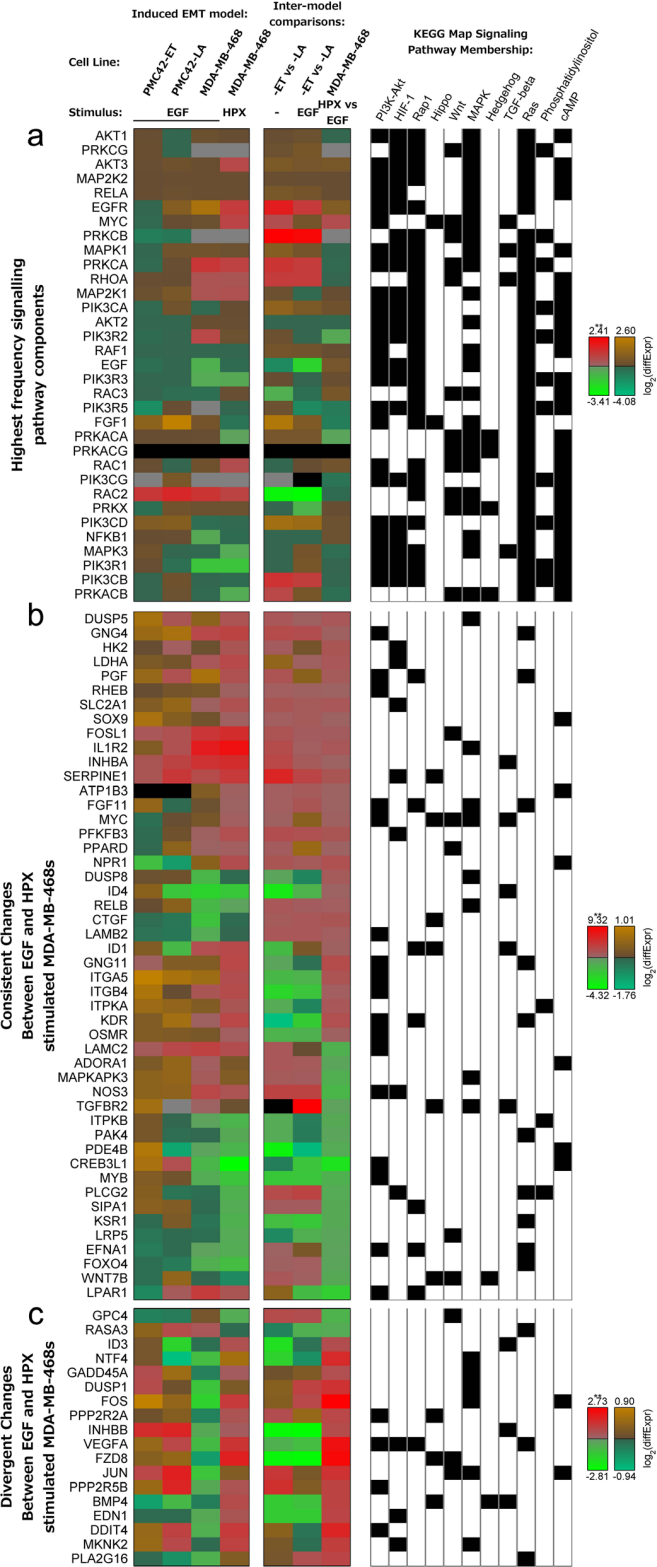
Numerous signalling components showed significant differences between EGF and HPX mediated EMT. Heat maps for: **(a)** mRNA transcripts for signalling components which are present across at least six perturbed signalling pathway KEGG maps (Table 1); **(b, c)** mRNA transcripts with significant (*q*-value < 0.05) differences in mRNA transcript abundance within at least one PMC42 cell line condition comparison, and differences in mRNA transcript abundance going in **(b)** the same, or **(c)** different directions for EGF or HPX-stimulated MDA-MB-468 cells compared to unstimulated, with a significant difference in transcript abundance between the EGF- and HPX-stimulated MDA-MB-468 cells. Membership within KEGG maps that are listed in Table 1 is shown at right (black box). Note the use of alternative colour-bars to indicate statistically significant (**; *q*-value < 0.05; *red-green*) and non-significant (*brown/orange*-*teal*) changes in abundance.

To identify common signalling elements across these different pathways we examined the frequency of components. Changes in mRNA transcript abundance of signalling proteins which were present within at least six of the 11 KEGG maps are shown in Figure 2a. Three proteins were found across seven pathways, encoded by: MAPK1, MAPK3 and PRKCA (Figure 2a; *see membership matrix at right*). Within six of the maps, the next most common proteins were encoded by: AKT1, AKT2, AKT3, MAP2K1, MAP2K2, PIK3CA, PIK3CB, PIK3CD, PIK3CG, PIK3R1, PIK3R2, PIK3R3, PIK3R5, PRKCB, PRKCG, and RAC1 (Figure 2a). The prevalence of MEK1/2-ERK1/2 and PI3K-Akt across these KEGG maps likely reflects the role of these signal transducers in the integration of numerous upstream signals.

### Systems-level computational analysis identified putative drug targets to alleviate signalling pathway dysregulation that occurs with induced EMT

As described earlier, EGF stimulation and hypoxic tumour environments are both thought to be clinically-relevant drivers of breast cancer progression *in vivo*. Thus, we focussed our analysis towards elucidating the convergent and divergent alterations to intracellular signalling which may encompass therapeutic targets for controlling EMT within MDA-MB-468 cells as a model of triple-negative breast cancer (TNBC).

To motivate drug target selection several analyses were performed and their results are described together below. First, transcripts showing similar or divergent patterns of differential expression between the EGF- and HPX-stimulated MDA-MB-468 cells were extracted (Additional file 3: Figure S2a & b, respectively). Components of the dysregulated signalling pathways (Table 1) are shown in Figure 2b & c. Transcripts that changed in the same direction across all models of induced EMT (including known EMT markers) are also shown in Additional file 4: Figure S3. Next, transcript abundance data were mapped onto an experimentally verified protein-protein interaction network and signalling components that could be targeted by drugs, inhibitors or antagonists were ranked by the relative level of dysregulation to their local interactome (Table 2).

**Table 2:** **Signalling pathway components showed variable levels of transcriptional disruption to their local interactome**

**Rank**	**HGNC symbol**	**degree**	**Rank**	**HGNC symbol**	**degree**	**Rank**	**HGNC symbol**	**degree**
1	HSP90AA1	164	11	PIK3R1	66	21	ERBB2	42
2	HSP90AB1	132	12	VCP	59	22	TGFBR1	41
3	EGFR	122	13	CALM1	58	23	HSPA1A	39
4	MYC	99	14	TUBB	57	24	RAC1	38
5	GSK3B	84	15	LYN	49	25	PIN1	37
6	FYN	76	16	JUN	48	26	NFKB1	37
7	ABL1	70	17	GAPDH	47	27	CDK6	35
8	PRKACA	68	18	FOS	45	28	MAPK3	35
9	PRKCA	67	19	CREBBP	45	29	TK1	34
10	CDK1	67	20	TUBA1A	42	30	PSMA7	34

mRNA transcripts encoding proteins for which there are drugs, inhibitors or antagonists available (through DrugBank v3.0), ranked by degree within a protein-protein interaction network of differentially expressed transcripts. Degree reflects the number of interaction partners (for the encoded protein) which show significant changes in transcript abundance.

**Figure 3:**
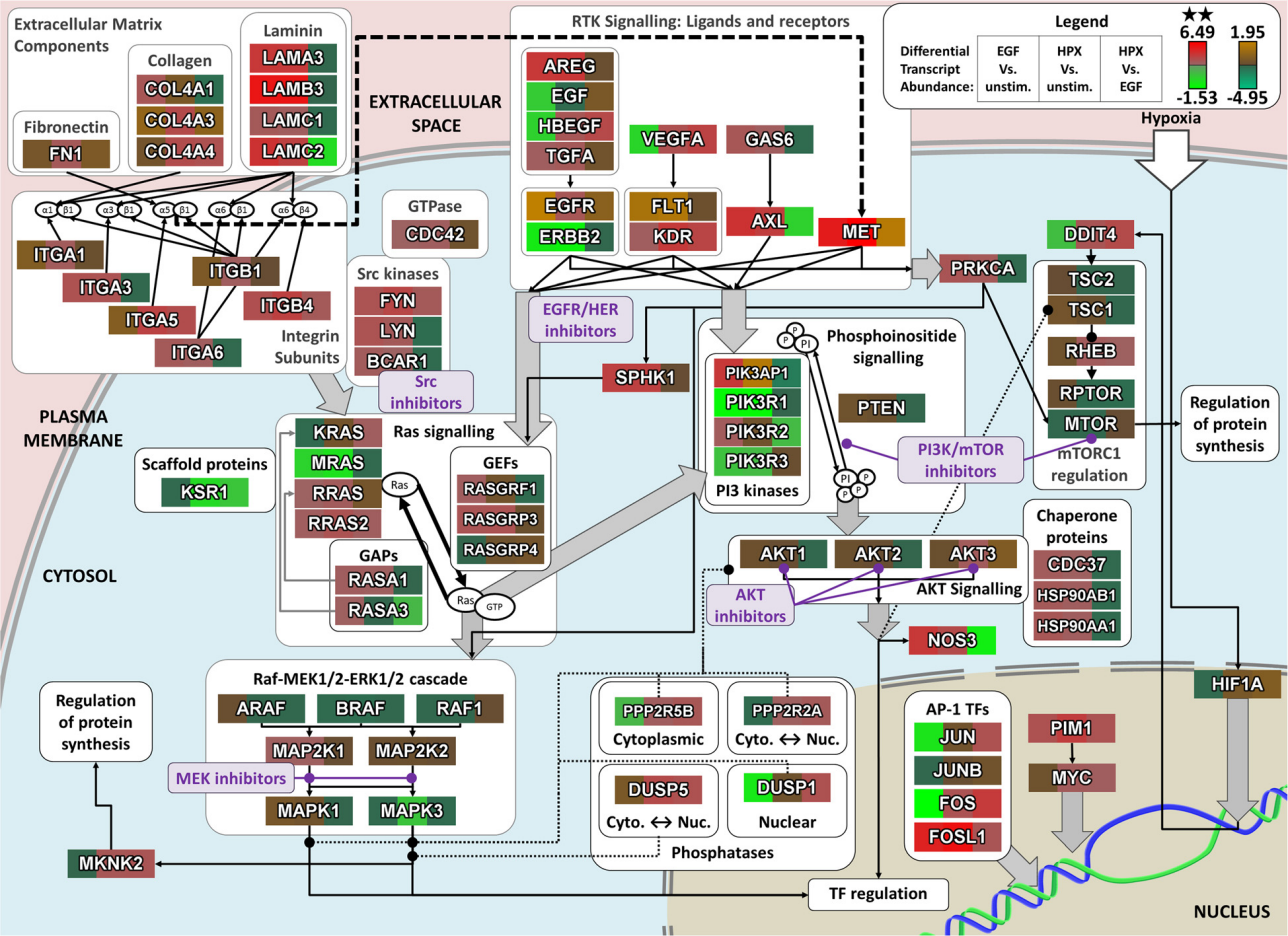
Differences in signalling component transcript changes between EGF and hypoxia induced EMT. Changes in transcript abundance (*legend top right*) for selected intracellular signalling components, within a schematic representation of the signalling network interactions between encoded proteins. Note the use of alternative colour-bars to indicate statistically significant (**; *q*-value < 0.05; *red-green*) and non-significant (*brown/orange*-*teal*) changes in abundance. Kinase inhibitors within the families selected for screening (*described in text*; *shown in purple*) are listed in Table 3.

These results are discussed below with a schematic diagram showing the role of proteins and functional relationships between signalling components, within the context of a broader intracellular signalling network (Figure 3). These results were used to motivate pharmacological targeting of several points within the dysregulated intracellular signalling network to examine the efficacy of blocking EMT, as indicated within Figure 3.

Given the use of EGF within our experimental models of *in vitro* induced EMT (Figure 1), kinase inhibitors against EGFR/HER were included as a positive control. The local interaction neighbourhoods of EGFR and ERBB2 were amongst the most dysregulated (Table 2); however, this may reflect the numerous feedback mechanisms which have previously been elucidated for EGFR signalling [62-64]. Alternative ligands for EGFR (TGFA, AREG and HBEGF) show significant changes in transcript abundance, suggesting that autocrine/paracrine signalling mechanisms may be activated, with HBEGF showing particularly strong differences between EGF and HPX stimulation (Figure 3).

Activation of EGFR is known to drive signalling through both PI3K-Akt and MEK-ERK [65], and these signal transduction cascades also appear to be key integrators across all of the dysregulated signalling pathways (Table 1; Figure 2a). Together with further details below, and the results of our pathway analysis, this motivated our experimental screening to focus upon different classes of kinase inhibitors targeting PI3K/mTOR, AKT and MEK1/2 as indicated (Figure 3).

Some of the strongest transcriptional changes with induced EMT were observed for integrin subunits and corresponding ECM components (Figure 3), and these changes would be expected to influence the formation and regulation of focal adhesion sites. Members of the Src kinase family play an important role in transducing signals from focal adhesion sites [66] to regulate Ras signalling, and the interactomes of both LYN and FYN are relatively enriched for disrupted binding partners, as is the homolog ABL1 (Table 2). Induction of FYN by PI3K/Akt signalling has previously been implicated as a key mediator of cell invasiveness [40]. LYN has also been identified as an important driver of phospho-tyrosine signalling to induce invasiveness within basal subtype breast cancers, although that study reported a relatively low level of activated LYN within the MDA-MB-468 cell line [67].

Pharmacological modulation of PI3K/mTOR was particularly attractive for this model of *in vitro* induced EMT, as MDA-MB-468 cells are known to carry an inactivating mutation in the PIP3-phosphatase PTEN [68]. Regulatory Class IA PI3K subunits stabilise the catalytic subunits to inhibit PI3K activity in the absence of upstream signals [69,70], and PIK3R1 (p85α) was significantly downregulated with EGF- or HPX-stimulation, although PIK3R2 (p85β) was increased, particularly with EGF stimulation (Figure 3). Furthermore, when considering disruption to the local interactome PIK3R1 was highly ranked, suggesting a greatly reduced threshold for signalling through PI3K, particularly under conditions where HPX is driving EMT.

Given the evidence for signalling through PI3K as described above, it was interesting to note changes in transcript abundance for the AKT scaffolding components Hsp90 and Cdc37 (Figure 3) with the HSP90AA1 and HSP90AB1 local networks showing the greatest degree of disruption (Table 2). Vivanco et al. demonstrated that GSK3B is an important downstream effector of AKT signalling [70], which also showed a high degree of disruption to the local interactome. Furthermore, AKT3 transcript abundance increased significantly under hypoxic conditions (Figure 3).

Increased signalling activity through MEK1/2-ERK-1/2 is the canonical downstream response to EGFR stimulation over many cell types [64,71], and activation of EGFR signalling induces a large number of feedback mechanisms to further modulate pathway activity [63]. This is consistent with the observation that MAPK3 (ERK1) showed some degree of disruption to its local interactome (Table 2), and with the notion that the MEK1/2-ERK-1/2 axis is a key integrator of dysregulated signalling pathways across the various models of induced EMT. It is possible that under conditions where key signalling proteins have been disrupted (*e.g.* an inactivating mutation in PTEN), some of these feedback mechanisms may lead to aberrant signalling. We examined differentially expressed genes with a previously identified transcriptional signature for MEK pathway activation [72] and found many of these transcripts were significantly upregulated within the EGF or HPX-stimulated MDA-MB-468 cells (Additional file 5: Figure S4a).

### EGF- and HPX-stimulated MDA-MB-468 cells show different responses to pharmacological inhibition of MEK-ERK and PI3K/Akt signalling

As detailed above, systems-level analysis of the mRNA transcript abundance changes that occurred with induction of EMT identified several signalling molecules that were likely to have dysregulated activity, and may play a role in promoting the mesenchymal phenotype. To investigate the potential for therapeutic intervention against these signalling components, a panel of kinase inhibitors (Table 3) was tested to determine the concentrations at which the fraction of vimentin^+^ cells or cell count was reduced by 50% (IC_50_ concentrations).

**Table 3:** **Targeted Inhibition of signalling molecules show differential effects between EGF- and hypoxia-induced EMT**

	**Cell line:**		**PMC42-ET**		**PMC42-LA**		**MDA-MB-468**			
	**Stimulus:**		**EGF**						**HPX**	
	**Measured IC**_**50**_ **for:**	**Biochemical assay (published data from compound vendors)**	**% vimentin-positive cells (μM)**	**Cell count (** **μM)**	**% vimentin-positive cells (** **μM)**	**Cell count (** **μM)**	**% vimentin-positive cells (** **μM)**	**Cell count (** **μM)**	**% vimentin-positive cells (** **μM)**	**Cell count (** **μM)**
**Targets:**	**Compound name**									
	Erlotinib (Tarceva)	EGFR (2 nM)	>25	15.04	0.18	16.42	0.14	16.42	5.71	>25
	Lapatinib (GW572016)	EGFR (10.2 nM), HER2 (9.8 nM)	>25	2.46	0.57	1.65	4.32	2.99	1.96	1.44
**a**) HERs/EGFR	Vandetanib (Zactima)	VEGFR2 (40 nM), VEGFR3 (110 nM), EGFR (500 nM)	>25	1.84	0.57	1.57	0.50	3.77	-	-
	Gefitinib (Iressa)	EGFR (33 nM)	>25	5.78	0.26	1.48	0.25	2.31	6.62	6.27
	TOVOK (Afatinib)	Irreversible binder. EGFR (0.5 nM), HER2 (14 nM)	>25	1.24	0.02	0.96	0.03	0.26	2.38	1.01
	AV-412	EGFR (43 nM), HER2 (282 nM)	>25	0.33	0.05	0.32	0.05	0.54	>25	0.06
	U0126	MEK1 (70 nM), MEK2 (60 nM)	>25	>25	1.38	>25	0.38	8.74	4.99	>25
	SL 327	MEK1 (180 nM), MEK2 (220 nM)	>25	16.43	1.43	>25	>25	12.56	>25	0.03
**b**) MEK-1/2	PD 198306	MEK (8 nM)	>25	1.36	0.34	2.50	0.46	0.97	1.7	2.94
	AZD6244 (Selumetinib)	MEK1 (14 nM)	>25	>25	0.06	>25	1.38	13.71	>25	>25
	CI-1040 (PD-184352)	MEK (1–1.3 nM)	>25	3.27	0.16	1.40	0.19	2.00	4.7	6.33
	PD0325901	MEK (0.33 nM)	>25	>25	<0.02	>25	<0.02	2.77	4.17	0.06
	PD173955-Analogue 1	c-Src (9 nM)	>25	5.94	>25	6.28	1.70	3.40	6.55	>25
	Saracatinib (AZD0530)	Src (2.7 nM)	>25	0.75	0.94	0.50	>25	6.35	>25	0.72
**c**) Src family	Bosutinib (SKI-606)	Src (1.2 nM), Abl (1 nM)	>25	1.40	0.23	1.17	0.25	1.05	1.19	0.54
	Dasatinib (BMS-354825)	Src (0.8 nM), Abl (0.6 nM)	>25	0.04	0.76	<0.02	0.64	5.59	>25	0.04
	PD173952	Src (8 nM), Lck (5 nM), FGFR1 (100 nM)	>25	0.35	1.61	0.23	0.10	0.25	-	-
	PIK-90	PI3K (α 11 nM, β 350 nM, γ 18 nM, δ 58 nM)	>25	16.35	>25	16.36	3.26	>25	>25	<0.02
	ZSTK474	PI3K (α 17 nM, β 53 nM, γ 6 nM)	>25	0.83	>25	2.11	0.21	0.72	2.35	>25
	GDC-0941	PI3K (α 3 nM, β 33 nM, γ 75 nM, δ 3 nM)	>25	0.46	8.67	1.18	1.27	1.79	4.69	4.88
	BEZ-235 (NVP-BEZ235)	p110 (α 4 nM, β 75 nM, γ 5 nM, δ 7 nM)	>25	0.06	>25	>25	0.02	0.05	0.05	3.43
**d**) PI3K/mTOR	PI103	DNA-PK (2 nM), mTORC1 (20 nM), PI3K-C2b (26 nM), p110 (α 8 nM, β 88 nM, γ 150 nM, δ 48 nM)	15.18	0.32	>25	1.82	0.38	0.88	1.22	1.04
	GNE-493	PI3K (α 3.4 nM, β 12 nM, γ 16 nM, δ 16 nM)	>25	1.11	>25	12.09	0.29	1.45	0.99	6.41
	GSK2126458 (HYR-582)	Ki: P110 (α 0.019 nM, β 0.13 nM, γ 0.06 nM, δ 0.024 nM), mTORC1 (0.18 nM), mTORC2 (0.3 nM)	>25	0.02	6.69	0.62	<0.02	0.08	0.29	0.74
	GNE-490	PI3K (α 3.5 nM, β 25 nM, γ 5.2 nM, δ 15 nM)	>25	2.16	>25	2.23	0.93	1.25	12.68	>25
	LY294002	PI3K (α 0.5 uM, β 0.97 uM, γ 0.57 uM)	>25	14.86	>25	12.12	4.18	13.22	>25	>25
	GSK690693	Akt1 (2 nM), Akt2 (13 nM), Akt3 (9 nM)	>25	>25	>25	8.32	>25	3.31	>25	>25
	A-674563	Ki: Akt1 (11 nM), PKA (16 nM), CDK2 (46 nM), ERK2 (260 nM)	>25	0.48	0.17	0.76	0.65	0.25	2.83	0.60
	Akt-i-1	Akt1 (4.6 μM)	>25	>25	>25	>25	>25	6.46	>25	12.30
**e**) Akt	Akt-i-1/2	Akt1 (58 nM), Akt2 (210 nM)	>25	>25	>25	>25	>25	2.82	>25	4.79
	AT7867	Akt1 (32 nM), Akt2 (17 nM), Akt3 (47 nM), PKA (20 nM)	>25	2.63	>25	0.24	>25	2.95	>25	4.57
	AZD5363	Akt1 (3 nM), Akt2 (8 nM), Akt3 (8 nM), ROCK2 (56 nM)	>25	>25	>25	>25	0.63	>25	>25	>25
	Merck-22-6	Akt1 (138 nM), Akt2 (212 nM)	>25	4.27	>25	1.48	>25	0.46	>25	0.55
	MK-2206	Akt1 (8 nM), Akt2 (12 nM), Akt3 (65 nM)	>25	5.62	>25	3.16	>25	1.86	>25	9.77

Inhibition of vimentin expression and cell counts by a selection of kinase inhibitors. Shown are the IC50 values (where the fraction of vimentin positive cells, or the cell count, was reduced by 50% compared to the controls). Concentrations are specified in μM units.

**Figure 4:**
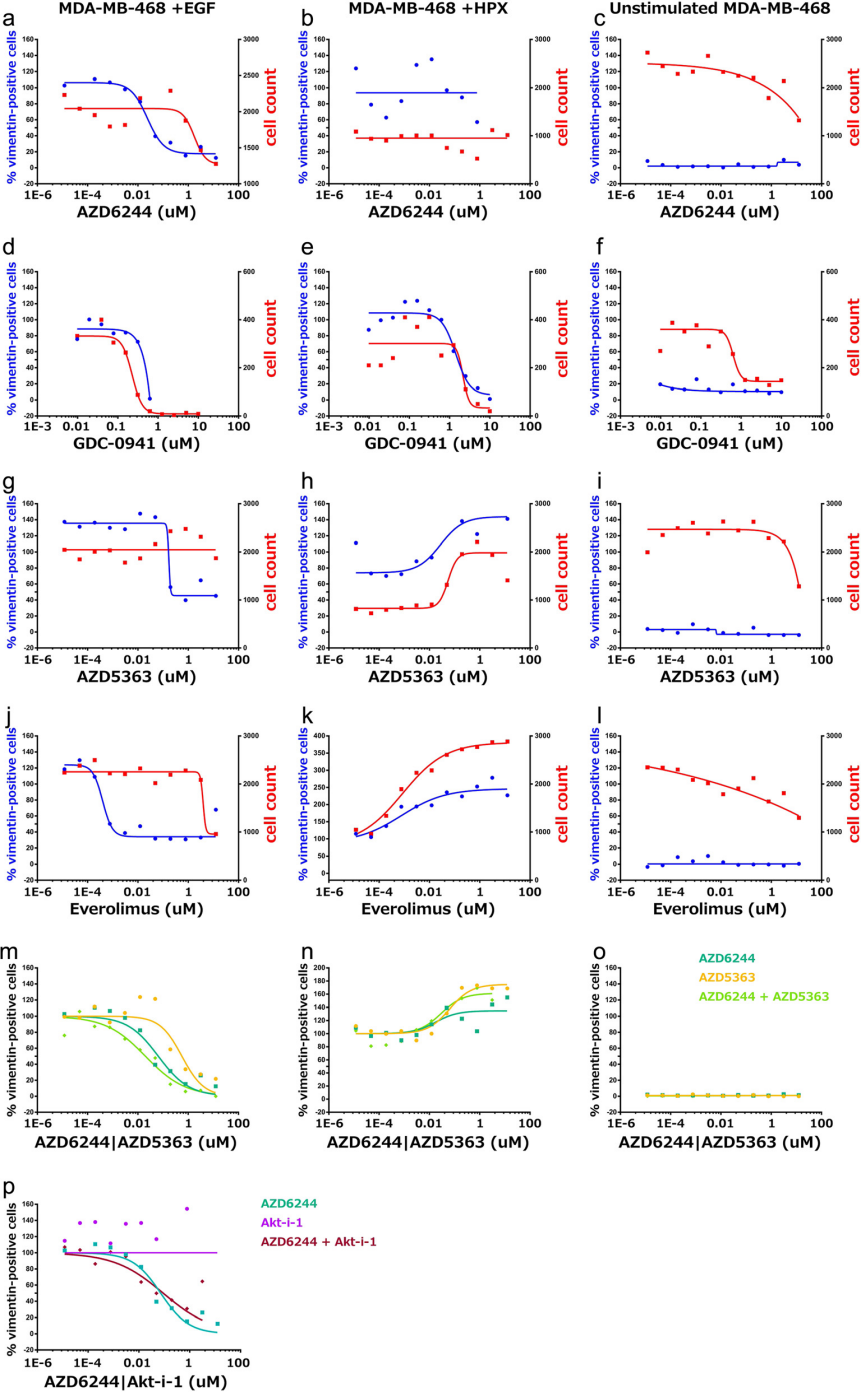
Hypoxia- and EGF-induced metastatic MDA-MB-468 cells show markedly different responses to pharmacological inhibitors. Pharmacological dose–response curves showing the fraction of vimentin-positive cells (*blue; left axes*) and cell-count (*red; right axes*) in the presence of **(a-c)** the MEK inhibitor AZD6244, **(d-f)** the PI3K inhibitor GDC-0941, **(g-i)** the AKT1/2/3 inhibitor AZD5363 **(j-l)** and the mTOR inhibitor Everolimus. **(m-o)** pharmacological inhibition of vimentin with a combination of MEK-1/2 (AZD6244) and AKT1/2/3 (AZD5363) inhibitors at varying concentrations. **(p)** pharmacological inhibition of vimentin with a comination of MEK-1/2 (AZD6244) and AKT1 (Akt-i-1) inhibitors at varying concentrations.

Inhibitors have been grouped according to the kinases they target. The dose–response curves for selected kinase inhibitors are shown in Figure 4. For reference, the IC50 values of each compound measured in biochemical assays with purified enzymes are included.

The majority of inhibitors tested on EGF-stimulated PMC42-ET cells were efficacious at reducing cell count; however, nearly every inhibitor tested had an IC_50_ for reducing the number of vimentin^+^ cells well above pharmacologically relevant concentrations (Table 3*a*-*e*), thus off-target effects are likely.

As expected, the panel of EGFR kinase inhibitors (Table 3*a*) were very effective at blocking EGF-induced EMT and cell growth in the PMC42-LA and MDA-MB-468 cells, and with the exception of lapatinib, the IC_50_ values for inhibition of vimentin expression are 8–10 fold lower than the corresponding IC_50_ values for reduction of cell count. Reduced levels of vimentin expression correlated with the ability of these compounds to inhibit the phosphorylation of ERK1/2 over a range of concentrations (Additional file 6: Figure S5), demonstrating the importance of the EGFR/MEK/ERK canonical pathway and its associated networks in promoting EMT-associated phenotypic changes. The EGFR kinase inhibitors also appeared to have an effect on HPX-stimulated MDA-MB-468 cells, although IC_50_ values for HPX-stimulated cells were all higher than corresponding IC_50_ values for EGF-stimulated cells. In particular, inhibition of the HPX-induced vimentin response in MDA-MB-468 cells occurred at drug concentrations >10-fold higher than required for inhibition of ERK phosphorylation, indicating that the MEK/ERK pathway may be less important for EMP and the regulation of vimentin expression under hypoxic growth conditions. This effect may also be due to drug resistance mechanisms as discussed below. EGFR kinase inhibitor-mediated reductions in cell count for both EGF- and HPX-stimulation were generally observed at concentrations an order of magnitude greater than the effects on vimentinexpression, indicating that our treatments are affecting EMT at relevant concentrations, while reduction in cell viability at higher concentrations may be caused by off target effects. Hypoxia-treated MDA-MB-468 cells were exposed to a small molecule inhibitor of HIF1α accumulation and gene transcriptional activity, CAY10585, to determine whether this could reduce the induction of EMT in these cells. At concentrations below 1 μM CAY10585 did not have a significant effect on the number of vimentin^+^ cells; however, the number of vimentin^−^ cells was potently reduced, suggesting this may have a deleterious effect upon the cell population with an epithelial phenotype (Additional file 7: Figure S6).

Although EGF stimulation further increased the mRNA transcript abundance of EMT markers (Figure 1*i*) the inability of EGFR inhibitors to reduce the fraction of vimentin^+^ PMC42-ET cells (Table 3a) suggests that the unstimulated mesenchymal phenotype of these cells is maintained through EGFR-independent signalling mechanisms.

Inhibitors targeting the MEK1/2 (Table 3*b*) and Src-family kinases (Table 3*c*) showed a similar response profile to the EGFR inhibitors with potent blocking of vimentin expression within the EGF-stimulated cells. For MEK inhibitors the IC_50_ values for inhibition of vimentin expression tended to be lower than the corresponding IC_50_ values for cell count, and within MDA-MB-468 cells the IC_50_ values were again higher with HPX stimulation than EGF stimulation (Table 3*b*). A similar effect was seen for inhibition of phospho-ERK1/2 (data not shown). A previously reported mRNA transcript signature for ‘compensatory resistance’ to AZD6244 (Additional file 5: Figure S4b) [72] shows some agreement with the observed efficacy of this MEK inhibitor (Table 3b). The EGF-stimulated PMC42-LA cells had the lowest IC_50_ for AZD6244 in reducing the fraction of vimentin^+^ cells by several orders of magnitude, and many of the AZD6244 resistance signature genes showed a decrease in transcript abundance relative to unstimulated PMC42-LA cells (Additional file 5: Figure S4b). Although the profile of this signature was very similar between EGF- and HPX-stimulated MDA-MB-468 cells, several of the transcripts showed a greater degree of upregulation with hypoxia, in agreement with the reduced efficacy of AZD6244 within hypoxia-stimulated MDA-MB-468 cells (Table 3b). For most Src family inhibitors within EGF-stimulated MDA-MB-468 cells the values for IC_50_ of vimentin^+^ cells were lower than the IC_50_ values for cell count. Conversely, within EGF-stimulated PMC42-LA cells and HPX-stimulated MDA-MB-468 cells the IC_50_ for cell count is lower for most Src family inhibitors (Table 3*c*).

Within EGF-stimulated PMC42-LA cells GDC-0941 and GSK2126458 were the only PI3K/mTOR inhibitors (Table 3*d*) with pharmacologically relevant IC_50_ values for reduction of vimentin^+^ cells, although most inhibitors were capable of reducing cell growth. The PI3K/mTOR inhibitors were much more efficacious within the MDA-MB-468 cells, and many of the tested compounds had a lower IC50 value for the reduction of vimentin^+^ cells compared to the reduction in cell count.

In contrast to PI3K/mTOR inhibitors, the majority of compounds targeting Akt kinases (Table 3*e*) were only capable of reducing cell count, with A-674563 and AZD5363 the only inhibitors with a pharmacologically relevant IC_50_ value for vimentin^+^ cells across any of the cell lines and conditions. Unexpectedly, some Akt inhibitors and mTOR inhibitors were observed to increase the fraction of vimentin^+^cells and the relative cell density, particularly within HPX-stimulated MDA-MB-468 cells (Figure 4h & k).

The observation that several classes of inhibitors were efficacious within EGF-stimulated MDA-MB-468 cells, but had little effect under hypoxic growth conditions, supports the conclusion from the transcriptome analysis that the phenotypically similar EMT processes induced with EGF or hypoxia are driven by different signalling mechanisms. Furthermore, given differences observed between the EGF- and HPX-induced transcriptional profiles, particularly for signalling ligands where the receptor also has strong increases in transcript abundance, such as HBEGF/EGFR and VEGFA/KDR, we hypothesised that pro-survival signalling through AKT may mediate the reduced efficacy of MEK-1/2 inhibitors under hypoxic conditions. Thus, we also applied the AKT1/2/3 inhibitors GSK690693 or AZD5363 in combination with the MEK1/2 inhibitor AZD6244. The pharmacological efficacy curves suggest that they provide a synergistic effect to block the relative fraction of vimentin^+^ cells (Figure 4 m). Furthermore, this effect was not observed with the AKT1 or AKT1/2 inhibitors tested in combination with AZD6244 (Figure 4p).

## Discussion

Transcriptional profiling of two human breast cancer models indicated that subtly different transcriptional responses underpinned EMT induced with EGF or HPX (Figure 1a-h &i). This included variation in the relative abundance of EMT-implicated transcription factors (Figure 1j & k), and alternative signalling pathways dysregulated by the transcriptional changes (Table 1 & 2; Figure 2 & 3). A panel of kinase inhibitors were selected from across the network of disrupted signalling components, within which PI3K-Akt and MEK1/2-ERK-1/2 appeared to act as signal integrators. In general, tested compounds had a much lower potency within our cellular assays compared to results obtained with purified enzymes. This was expected due to effects such as competition with high levels of intracellular ATP, binding to other proteins and limited cellular permeability [73].

A range of factors regulate EMP through various signalling pathways [74], and “kinase switching” from the ErbB axis to FGFR and PDGFR has been associated with EMT in NSCLC models [75]. We focussed on differences in the signalling mechanisms associated with EGF- or HPX-induced EMT within MDA-MB-468 cells as a model of TNBC (Figure 4). Many drugs that induced a response within the EGF-stimulated MDA-MB-468 cells, such as the MEK inhibitor AZD6244 (Figure 4a & b), showed reduced efficacy or even pro-proliferative effects within HPX-stimulated MDA-MB-468 cells. It has previously been reported that hypoxia can have varied effects across different kinase inhibitors, and this may be partially mediated by modulation of hypoxia-induced compensatory mechanisms, such as VEGF signalling [76]. Stark differences were observed in the responses elicited by some inhibitors, including the mTOR inhibitor Everolimus, and the AKT1/2/3 inhibitor AZD5363 (Figure 4 g & h; j & k). These divergent responses to pharmacological perturbation support the conclusion that subtly different signalling mechanisms are responsible for driving the phenotypically similar EMT processes that occurred with EGF or HPX stimulation of MDA-MB-468 cells (Figure 1). Intriguingly, synergistic effects for blocking EGF-induced EMT were observed when combining an AKT1/2/3 inhibitor with the MEK-1/2 inhibitor AZD6244, but not for an inhibitor which targeted AKT1 alone (Figure 4p), indicating that AKT1 is not solely responsible for the protective signalling seen in this system.

### Differences in the transcriptional profile and pharmacological responsiveness between EGF- and hypoxia-induced EMT

We saw differences in the transcript abundance and/or regulation of several well-studied TFs previously associated with EMT in breast cancer [77-79] (Figure 1j). Relatively large (although not statistically significant) changes in transcript abundance for TWIST1 under HPX conditions are consistent with its reported regulation by HIF1 [36,37,80], as are the increases for ZEB1 [80] (Figure 1j). Failure to detect SNAI2 transcripts within the MDA-MB-468 cells data was consistent with one previous report [81]; however, increases in SNAI2 mRNA abundance following EGF stimulation of MDA-MB-468 cells have been reported together with enrichment at sites of *in vivo* EMT [48]. This discrepancy may reflect the different provenance of MDA-MB-468 cells in Belgium and Australia, or alternatively, SNAI2 transcripts may be expressed at sufficiently low levels that they approach the signal-to-noise ratio of our RNA-Seq protocol. Our previous study showed that treatment of MDA-MB-468 cells with HPX caused a non-significant increased at different time points in SNAI2, TWIST1 and ZEB2, and a significant increase in ZEB1 [42]. The dominant role of ZEB1 in the current study is also consistent with our previous observations that PMC42-ET cells have significantly higher levels of ZEB1 and SNAI2 than PMC42–LA cells, and that ZEB1 and SNAI2 were both increased in PMC42-LA cells after EGF treatment [41,42]. ZEB1 appears to be a downstream integration point for EMT regulation [41], and is subject to complex regulation at multiple levels [82,83]. This differential control between EMT scenarios may have clinical utility in allowing selective inhibition of EMT mechanisms involved in tumour progression, whilst leaving critical physiological processes unperturbed to reduce toxicity.

Numerous transcripts showed differences in abundance between EGF- and HPX-mediated EMT (Figure 2 & 3), and some of the altered signalling components likely contributed to the variable drug efficacy. The multidrug resistance-promoting P-glycoprotein (ABCB1) had a large increase in transcript abundance within hypoxia-stimulated MDA-MB-468 cells (2.6-fold increase, *q*-value = 0.029; for EGF-stimulation 1.6 fold increase, *q*-value = 0.36), which may have reduced the efficacy of several kinase inhibitors [76,84,85].

The ability of EGFR inhibitors to block EMT in HPX-stimulated MDA-MB-468 s (albeit at higher concentrations than in EGF-stimulated MDA-MB-468 s; Table 3a) suggests that hypoxia-induced EMT may be partially mediated by paracrine/autocrine EGFR signalling. This is supported by the observation that transcript abundances for several EGF ligands were significantly increased with HPX stimulation, as was EGFR itself (Figure 3). This could drive paracrine/autocrine EGFR signalling to promote EMT, although it should be noted that EGF was present within the culture media and this would also drive some EGFR signalling. HB-EGF mediated activation of EGFR is an important driver of MDA-MB-231 cell invasiveness, particularly for brain metastases [86]. A similar role has been described for autocrine TGFβ signalling in promoting EMT [82], and it is interesting that modulators of TGFB signalling showed significant changes in transcript abundance, including: THBS1 (Figure 1i), INHBA, TGFBR2 (Figure 2b), INHBB and BMP4 (Figure 2c).

Many of the ‘HIF-1 signalling’ transcripts with large differences in abundance between EGF- and HPX-stimulated MDA-MB-468 cells were known transcriptional targets of HIF-1, such as SERPINE1, VEGFA, and EDN1. Although it is not included within the KEGG HIF-1 signaling pathway, DDIT4 is a known HIF-1-responsive transcript (HIF-1 responsive RTP801) which can modulate mTORC1 through the RHEB inhibitors TSC1/TSC2. Other HIF-1 target genes necessary for metabolic adaptation to hypoxic growth also showed large differences, including HK2, LDHA, PFKFB3, and SLC2A1 (Figure 2b). These differences likely reflect stabilisation of HIF-1α under hypoxic growth conditions, although many of the metabolism-associated HIF-1 targets also had increased transcript abundance with EGF stimulation (Figure 2b). This may be mediated by MEK1/2-ERK1/2 signalling through MKNK2 to eIF4E, influencing HIF-1α translation. Pre-clinical studies have observed such effects with hypoxia-induced EMT [39] and this may contribute to the deleterious effect of VEGFR inhibitors [87].

Assuming that increased abundance of SOX9 mRNA (Figure 1i) contributes to increased transcription factor activity, this driver of mammary stem cell behaviour likely promotes EMT (Figure 1a–h). The increase in SOX9 transcript abundance was only significant within the HPX-stimulated MDA-MB-468 cells (Figure 1i), although SOX9 also showed strong differences in transcript abundance (*q*-value < 0.05) between the ET and LA sublines (Figure 2c). SOX9 has been linked to clinical chemoresistance in colorectal cancer [88], an affect which may be partially mediated by EMT changes. Furthermore, cancer stem cell markers are negative predictive markers for the efficacy of everolimus in treating TNBC [89], and this may underpin the failure of everolimus to block EMT within hypoxia-stimulated MDA-MB-468 cells (Figure 4 k).

### Pathway convergence on PI3K-Akt and MEK1/2-ERK1/2

The presence of PI3K-Akt and MEK1/2-ERK1/2 components across multiple signalling pathways (Figure 2a) is consistent with the role of these evolutionarily-conserved modules in the integration of various extracellular stimuli [90]. As detailed above, PI3K-Akt and MEK1/2-ERK-1/2 are well-known effectors of EGFR signalling [65] and other receptor tyrosine kinases [69,90], and signalling through these pathways can induce a variety of feedback mechanisms [63]. Even within breast cancer cell lines that do not over-express HER2, such as triple negative MDA-MB-231 cells, EGFR-induced signalling through PI3K/Akt is thought to be involved in mediating EMT [31], while several other studies implicate MEK1/2-ERK1/2 signalling as an important driver [91]. The relatively high frequency of PI3K-Akt and MEK1/2-ERK1/2 may, simply reflect their relatively well-studied nature, however, leading to inclusion across numerous KEGG maps.

### Activation of MEK-ERK signalling and promotion of signalling through ERK2 may contribute to EMT development in hypoxia

Recent evidence has indicated that ERK2 (MAPK1) signalling is central to EMT, activating DEF-motif transcription factors such as FOSL1 (FRA1) and ZEB1/ZEB2 [92,93]. Although increases in ERK2 (MAPK1) transcript abundance were not significant with EGF or HPX stimulation, there were corresponding decreases in ERK1 (MAPK3) transcript abundance that were significant within hypoxia-stimulated MDA-MB-468 cells (Figure 3). The altered ratio of transcripts may have led to ERK2 becoming the dominant isoform, while activation of Ras under stimulated conditions drives signalling though MEK-1/2 (Additional file 5: Figure S4a) to phosphorylate ERK2. Kinome profiling of TNBC tumours suggests that ERK2 is activated compared to control tissue, while ERK1 activity remains unchanged [94], and it is tempting to speculate that the hypoxic tumour environment drives *in vivo* ERK2 activation. FOSL1 was one of the few transcripts significantly upregulated across all our models of induced EMT and ZEB2 was not expressed within MDA-MB-468 cells (Figure 1j), suggesting ZEB1 and FOSL1 may be sufficient to mediate this transformation.

### Clinical Implications

Oncogenic mutations of Ras are important drivers of malignant behaviour within melanoma and pancreatic cancers, and although such activating mutations are relatively rare within breast cancer, overexpression of Ras mRNA and protein has been demonstrated [95]. Our data show a strong increase in transcript abundance for RRAS2 (Figure 3), consistent with reports that RRAS2 drives PI3K-dependent tumorigenesis and contributes to late stage metastasis in certain lung cancers [96]. Activation of Ras proteins by a range of growth factor receptors [97] leads to activation of the Raf-MEK-ERK [94] and the PI3K-Akt signal transduction cascades, culminating in the regulation of cellular survival and proliferation genes [90,98]. Ras is difficult to target therapeutically [99], although up- and downstream pathway components may be inhibited [100]. Inhibition of Src upstream has proved disappointing with response rates below 10% in unselected TNBCs [101] whereas downstream B-Raf inhibition is currently unexplored.

The effects of MEK inhibition within breast cancer are poorly studied in comparison to other cancers, particularly melanoma and lung cancer. Treatment of MDA-MB-231 and SUM159 cells with the MEK inhibitor AZD6244 has been shown to reduce c-Myc mRNA transcript and protein abundance, leading to receptor tyrosine kinase reprogramming which drives MEK inhibitor resistance [94]. Breast cancer cell lines sensitive to the MEK inhibitor selumetinib tend to be a basal subtype with Raf mutations [102], and a number of MEK inhibitors are in early clinical trials across solid tumour types, although information on breast cancer responsiveness is still scarce [103]. A phase II clinical trial for the MEK inhibitor CI-1040 in chemotherapy pre-treated metastatic breast cancer showed no major effects, although one patient developed stable disease [104]. The lack of frequent mutations within the core Ras-Raf-MEK axis, but the potential for cross-talk with a plethora of pathways intrinsic to breast cancer progression, may mean that the potential of MEK blockade lies in treatment combinations to overcome resistance. This is borne out by pre-clinical studies which have shown MEK inhibition has the potential to enhance sensitivity of breast cancer xenografts to HER2 blockade [105] and anti-estrogen treatment [106]. Furthermore, studies with breast cancer cell lines have shown that MEK inhibition also increases sensitivity to EGFR blockade [107], and reversed the effects of IGF-1R overexpression in promoting proliferation [108].

Combination therapies with PI3K inhibition have been shown to enhance the effect of MEK inhibition within basal subtype breast cancer cells by alleviating the compensatory activation of PI3K/AKT that occurs with MEK inhibition [109]. We observed that a combination of MEK1/2 and AKT1/2/3 inhibitors had synergistic effects in blocking vimentin induction within our *in vitro* model of EGF-induced EMT (Figure 4m). It should be noted, however, that when EMT was induced with hypoxic growth conditions this combination of kinase inhibitors promoted an increase in the relative fraction of mesenchymal cells (Figure 4n) demonstrating the importance of elucidating the detailed effects of pathway manipulation in this area before proceeding to clinical studies. Depletion of AKT3 has previously been reported to sensitize TNBC cell lines, including MDA-MB-468 cells, to the pan-Akt inhibitor GSK690693 [110] and The Cancer Genome Atlas Project (TCGA) data show that AKT3 is upregulated in 28% of TNBCs [110,111]. The observation that inhibitors targeting AKT1 (Figure 4p) or AKT1/2 (data not shown) did not have a combinatorial effect with MEK1/2 inhibition suggests that AKT3 mediates sufficient signal transduction to provide functional compensation during inhibition of AKT1 and AKT2. This specific possibility remains to be tested.

## Conclusions

In this report we have studied the mRNA transcript profile across several models of induced EMT to identify common dysregulated signalling components that may contribute to development and maintenance of the metastatic phenotype. Given the putative role for EGF signalling and hypoxia in mediating tumour progression *in vivo*, our analysis focussed on the differences between these stimuli in promoting EMT within MDA-MB-468 cells as a model of triple negative breast cancer. A number of kinase inhibitors were targeted at different points across the network of dysregulated signalling components, and the alternative stimuli were associated with variation in the efficacy of kinase inhibitors at blocking induction of EMT. A combination of AKT1/2/3 and MEK1/2 inhibitors was shown to have synergistic effects on blocking the induction of EMT *in vitro.* The effects of simultaneously blocking these important signalling pathways are likely to be deleterious to many different cell types; however, using novel targeted drug delivery mechanisms that are under development it may be possible to apply this combination therapy for the clinical treatment of EMT within TNBC. Furthermore, with further comparative study the differential control of EMT by alternative driver molecules we have identified may allow a selective effect to be exerted on pathogenic EMT processes whilst leaving physiological processes intact, thereby minimising toxicity to patients.

We have demonstrated that hypoxic conditions fundamentally change the way breast cancer cells respond to drugs and compounds in various stages of development for treatment of breast cancer. The role of HIF1 in promoting a mesenchymal phenotype has been well explored [4,11,36,37], as has the role of hypoxia in promoting drug resistance components [76,84,85]. Perhaps most importantly, our data also indicate that under hypoxic conditions some therapeutic interventions may shift cells into an even more aggressive, mesenchymal phenotype. This highlights the vital importance of evaluating novel drug targets under a more-representative range of stimuli and conditions that mimic the heterogeneity of environmental conditions tumours are exposed to *in situ*. While attention has recently been drawn to the problem of genetic heterogeneity in breast cancer tumours, our study indicates that extra-cellular conditions, such as those we have explored here to stimulate EMT, can induce divergent molecular states even on a common genetic background, resulting in altered drug sensitivity and response. Given the hypoxic conditions commonly prevalent in the core of solid breast tumours, these findings have clear clinical implications for both treatment, and the drug development process.

## Additional files

Additional file 1: Table S1. Provenance of EMT-associated transcripts in Figure 1j. References which describe the role of transcripts listed Figure 1j in promoting EMT. Additional file 2: Figure S1. mRNA abundance data of selected targets with comparison to published data sources for selected transcription factors. (**a**) changes in mRNA transcript abundance (for Figure 1i & 1j transcripts) between specified cell lines and conditions (*at top*), with the mean count value (normalised to counts-per-million; CPM) of the compared conditions overlaid. Note the use of alternative color-bars to indicate statistically significant (**; *q*-value < 0.05; *red-green*) and non-significant (*brown/orange*-*teal*) changes in abundance. Black squares indicate mRNA transcripts that were not reliably detected. (**b**) Differential transcript abundance within and between the *in vitro* models of induced EMT for Blick Basal B discriminator transcripts [13]. Note the use of alternative color-bars to indicate statistically significant (**; *q*-value < 0.05; *red-green*) and non-significant (*brown/orange*-*teal*) changes in abundance. Black squares indicate mRNA transcripts that were not reliably detected. (**c**) Relative transcripts abundances for EMT implicated TFs (from Additional file 2: Figure S1a) within the Heiser data [112]. MDA-MB-468 cells are highlighted (cyan). (**d**) Relative transcripts abundances for EMT implicated TFs (from Additional file 2: Figure S1a) within the Neve data [44]. MDA-MB-468 cells are highlighted (cyan). Unstimulated MDA-MB-468 cells (Figure 1 & Additional file 2: Figure S1a) have a relatively high abundance of SOX9 and a relatively low abundance for FOXA2, FOSL1 SNAI2, TWIST1, ZEB1, ZEB2, in agreement with the both the Heiser et al. [112] (Additional file 2: Figure S1c) and the Neve et al. [44] (Additional file 2: Figure S1d) studies. Additional file 3: Figure S2. Changes in abundance for selected mRNA transcripts. Heat maps (*legend at left*) showing changes in transcript abundance between the specified conditions (*at top*) for specified transcripts where there is a significant difference in abundance between EGF and HPX stimulated MDA-MB-468 cells, with (**a**) divergent or (**b**) consistent changes between EGF and HPX stimulated MDA-MB-468 cells. Targets have been clustered by expression pattern and are ordered accordingly. Additional file 4: Figure S3. Changes in abundance for selected mRNA transcripts. Transcripts with consistent differential transcript abundance across all condition comparisons, and their membership across all KEGG maps (*at right*). Additional file 5: Figure S4. Components of transcriptional signatures for MEK signalling activation (*at top*) and compensatory resistance to AZD6244 (*at bottom*). To examine the hypothesis that signalling through MEK is implicated in these models of induced EMT, we examined changes in the abundance of genes previously classified as ‘transcriptional signatures’ for MEK pathway activation and AZD6244 sensitivity [72]. A large number of transcripts within the MEK signalling activation signature showed increased transcript abundance in the EGF stimulated PMC42-ET and PMC42-LA cells, with most of the changes not being statistically significant. Although changes in transcript abundance for components of the MEK signalling signature are less consistent within the stimulated MDA-MB-468 s, there were more transcripts showing a significant (*q*-value < 0.05) increase in abundance suggesting that signalling through MEK1/2 is active. Examining mRNA transcripts which have been associated with compensatory resistance to AZD6244, the EGF stimulated PMC42-LA cells show a number of transcripts with decreased abundance. For the EGF and HPX stimulated MDA-MB-468 cells a number of these transcripts show increased abundance, with more than half showing a statistically significant (*q*-value < 0.05) increase in abundance within MDA-MB-468 cells grown under hypoxic conditions. It is interesting to note that the PMC42-ET and –LA subline comparison showed the weakest signature for MEK signalling activation, and the strongest signature for AZD6244 resistance, regardless of EGF stimulation. The AZD6244 resistance signature shows relatively good agreement with our inhibitor screen results (Table 3), such that EGF stimulated PMC42-LA cells are susceptible to AZD6244, the inhibitor is slightly less efficacious within EGF stimulated MDA-MB-468 cells, and MDA-MB-468 cells grown under hypoxic conditions are not affected by AZD6244. Additional file 6: Figure S5. Inhibition of ERK phosphorylation shows good correlation with the inhibition of vimentin-positive cells within EGF and HPX induced EMT. Pharmacological response curves for the MEK-1/2 inhibitor CI-1040, showing the %inhibition of vimentin (*blue*), %inhibition of phospho-ERK-1/2 (*green*) and reductions in cell count (*red*). (**a**) Note that there is good correlation between the inhibition of vimentin and phospho-ERK over a range of concentrations for EGF stimulated MDA-MB-468 cells. (**b**) For MDA-MB-468 cells grown under hypoxic conditions, inhibition of phospho-ERK shows a similar response; however, inhibition of vimentin expressing cells only occurs at relatively high concentrations of phospho-ERK, suggesting the activation of compensatory signalling mechanisms. Additional file 7: Figure S6. Within hypoxia-treated MDA-MB-468 cells, inhibition of HIF1α with the small molecule inhibitor CAY10585 or transfection with siRNA targeting HIF1α caused a large decrease in the number of vimentin-negative cells. Hypoxia-treated MDA-MBA-468 cells were treated with the small molecule inhibitor of HIF1α activation and nuclear accumulation, CAY10585, or vector/negative-control (DMSO). Relative to the DMSO treated cells, CAY10585 caused no significant change in the cell count of vimentin^+^ cells; however, there was a profound reduction in the number of vimentin^−^ cells. To ensure that the effect is target-related, an siRNA targeting HIF1α was tested, together with a scrambled siRNA/negative-control. Relative to the scrambled siRNA treatment, the HIF1α siRNA caused a small increase in the number of vimentin^+^ cells; however, there was a much larger reduction in the number of vimentin^−^ cells, in concordance with the inhibitor data. The transfection of siRNA into MDA-MB-468 cells was performed as a reverse transfection. Lipofectamine 2000 (Life technologies, 0.25 uL per well) and siRNA (final concentration 40 nM) were separately diluted into 25 uL of Opti-MEM (Life technologies) and incubated for 5 minutes. The solutions were then combined and incubated for 15 minutes at room temperature. To this mixture, 8,000 cells diluted into 100 uL of DMEM with 10% FBS, and the combined mixture was seeded in a 96 well assay plate (Corning, #3603). To other wells, 8,000 MDA-MB-468 cells were seeded without any siRNA or transfection reagent. The plates were incubated over night at 37C/5% CO2. The next day, media in the transfected wells were replaced with fresh media. HIF1a inhibitor (CAY10585, sc-205346, Santa Cruz biotechnology) was diluted in DMSO (0.5% final concentration) and added to the untransfected cells at the concentrations indicated in the figure. As a control, cells were treated with 0.5% DMSO. All siRNA and inhibitor reactions were performed in triplicate. The cells were incubated in a hypoxia chamber (1% O2) for 72 h, before being fixed with 3.7% formaldehyde for 15 minutes. The cells were then stained for Vimentin, imaged and analysed as previously described. The siRNAs used were HIF1a siRNA (sc-35561, Santa Cruz Biotechnology) and scrambled control siRNA (sc-37007, Santa Cruz Biotechnology). Additional file 8: Table S2. Estimated *p*-values for enrichment of elements within KEGG signal transduction maps without correction for multiple hypothesis testing. Signaling pathway KEGG maps which are over-represented (*p*-value < 0.05) for transcripts which show differential expression across at least one of the specified conditions are listed in Table 1.

## Abbreviations

BCSC: Breast cancer stem cell
BrCa: Breast cancer
EGF: Epidermal growth factor
EMP: Epithelial-mesenchymal plasticity
EMT: Epithelial-mesenchymal transition
ERK: Extracellular-signal regulated kinase
HPX: Hypoxia
MAPK: Mitogen activated protein kinase
MaSC: Mammary stem cell
MEK: MAPK/ERK kinase
MET: Mesenchymal-epithelial transition
PI3K: Phosphatidyl-inositol 3 kinase
TBS: Tris-buffered saline
TF: Transcription factor
TNBC: Triple-negative breast cancer
