# Supplementary figures and images for: Stimulus-dependent differences in signalling regulate epithelial-mesenchymal plasticity and change the effects of drugs in breast cancer cell lines
Source: Cell Commun Signal. 2015 May 15;13:26. doi: 10.1186/s12964-015-0106-x (PMC4432969; doi:10.1186/s12964-015-0106-x)

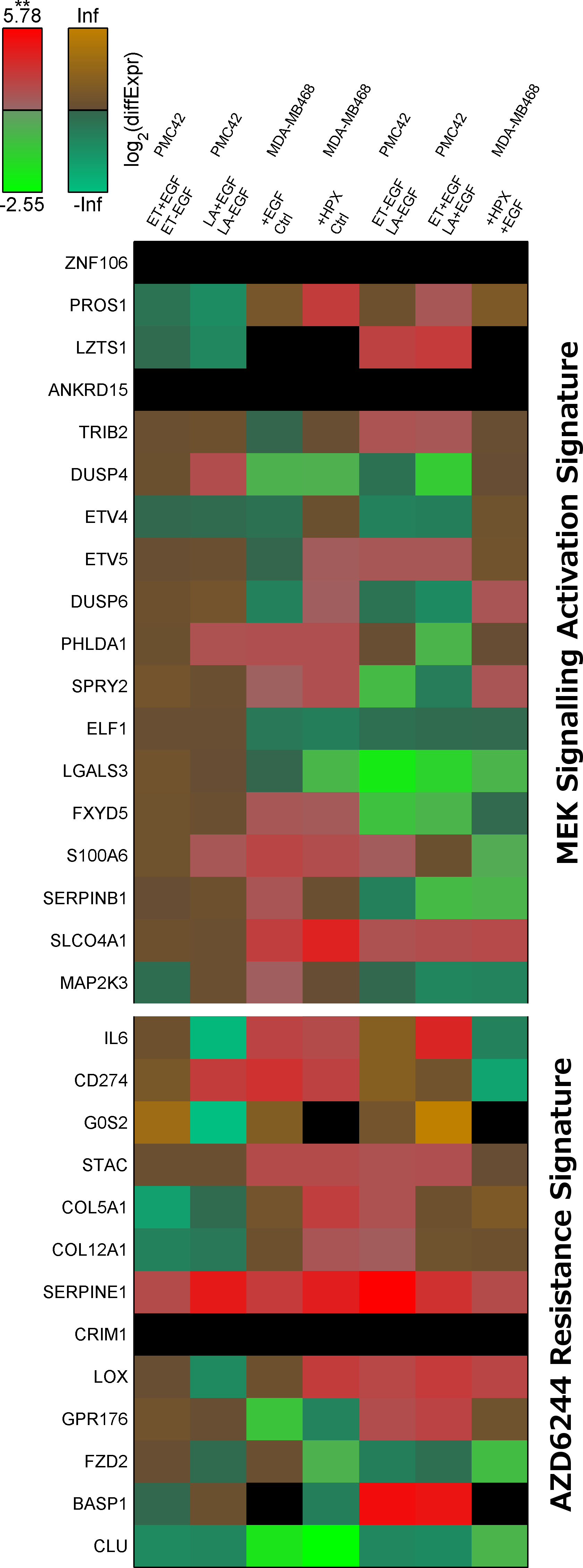

Supplement: Additional file 5: Figure S4. — Components of transcriptional signatures for MEK signalling activation (at top) and compensatory resistance to AZD6244 (at bottom). To examine the hypothesis that signalling through MEK is implicated in these models of induced EMT, we examined changes in the abundance of genes previously classified as ‘transcriptional signatures’ for MEK pathway activation and AZD6244 sensitivity [72]. A large number of transcripts within the MEK signalling activation signature showed increased transcript abundance in the EGF stimulated PMC42-ET and PMC42-LA cells, with most of the changes not being statistically significant. Although changes in transcript abundance for components of the MEK signalling signature are less consistent within the stimulated MDA-MB-468 s, there were more transcripts showing a significant (q-value < 0.05) increase in abundance suggesting that signalling through MEK1/2 is active. Examining mRNA transcripts which have been associated with compensatory resistance to AZD6244, the EGF stimulated PMC42-LA cells show a number of transcripts with decreased abundance. For the EGF and HPX stimulated MDA-MB-468 cells a number of these transcripts show increased abundance, with more than half showing a statistically significant (q-value < 0.05) increase in abundance within MDA-MB-468 cells grown under hypoxic conditions. It is interesting to note that the PMC42-ET and –LA subline comparison showed the weakest signature for MEK signalling activation, and the strongest signature for AZD6244 resistance, regardless of EGF stimulation. The AZD6244 resistance signature shows relatively good agreement with our inhibitor screen results (Table 3), such that EGF stimulated PMC42-LA cells are susceptible to AZD6244, the inhibitor is slightly less efficacious within EGF stimulated MDA-MB-468 cells, and MDA-MB-468 cells grown under hypoxic conditions are not affected by AZD6244. [file 12964_2015_106_MOESM5_ESM.png]

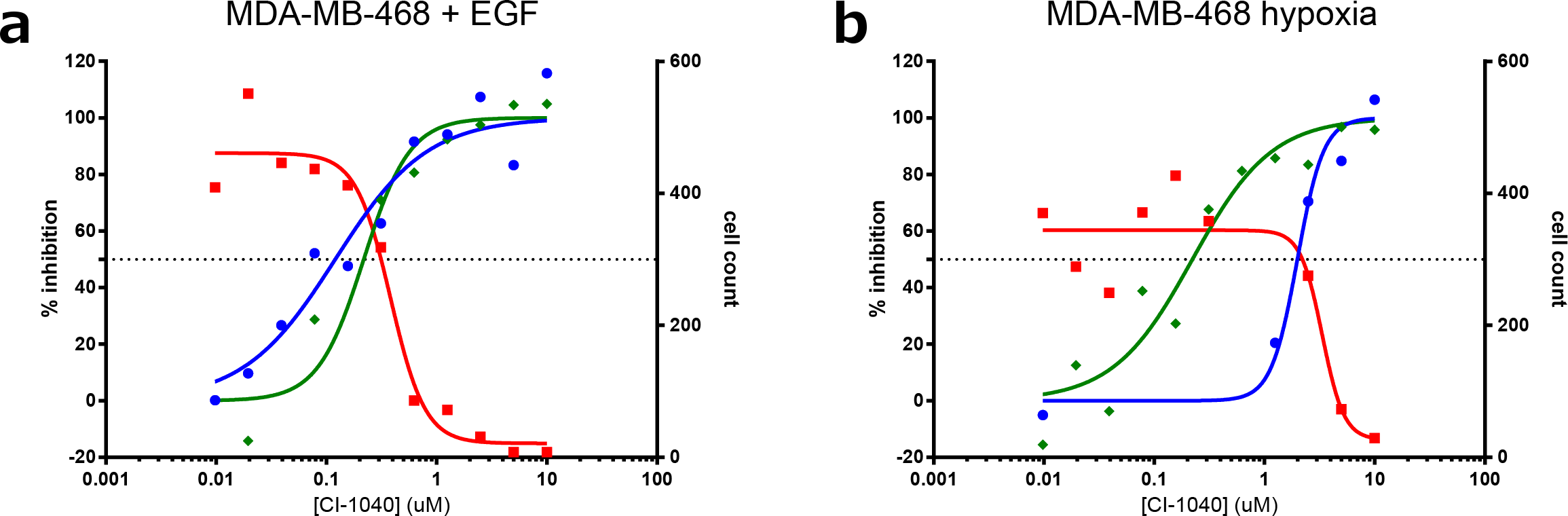

Supplement: Additional file 6: Figure S5. — Inhibition of ERK phosphorylation shows good correlation with the inhibition of vimentin-positive cells within EGF and HPX induced EMT. Pharmacological response curves for the MEK-1/2 inhibitor CI-1040, showing the %inhibition of vimentin (blue), %inhibition of phospho-ERK-1/2 (green) and reductions in cell count (red). (a) Note that there is good correlation between the inhibition of vimentin and phospho-ERK over a range of concentrations for EGF stimulated MDA-MB-468 cells. (b) For MDA-MB-468 cells grown under hypoxic conditions, inhibition of phospho-ERK shows a similar response; however, inhibition of vimentin expressing cells only occurs at relatively high concentrations of phospho-ERK, suggesting the activation of compensatory signalling mechanisms. [file 12964_2015_106_MOESM6_ESM.png]

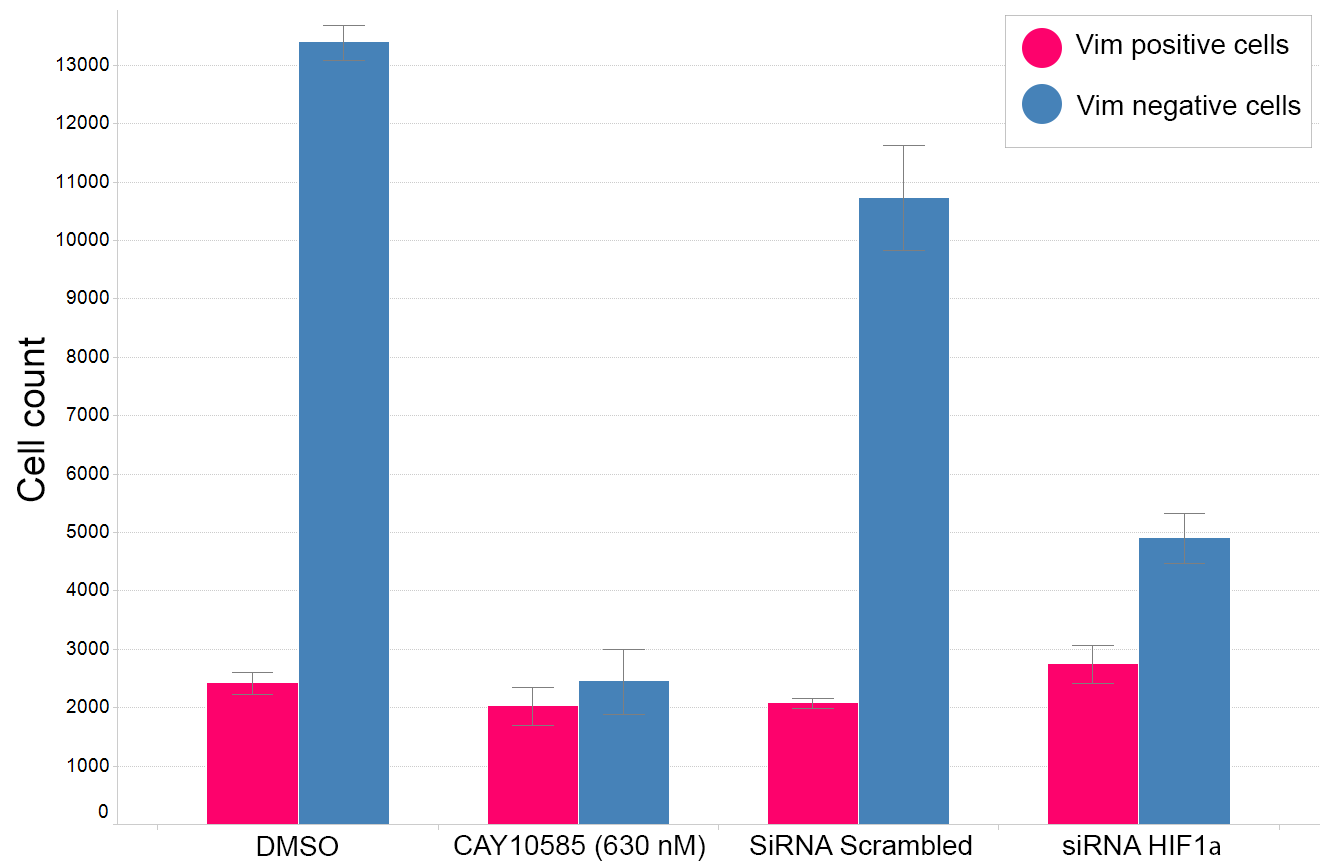

Supplement: Additional file 7: Figure S6. — Within hypoxia-treated MDA-MB-468 cells, inhibition of HIF1α with the small molecule inhibitor CAY10585 or transfection with siRNA targeting HIF1α caused a large decrease in the number of vimentin-negative cells. Hypoxia-treated MDA-MBA-468 cells were treated with the small molecule inhibitor of HIF1α activation and nuclear accumulation, CAY10585, or vector/negative-control (DMSO). Relative to the DMSO treated cells, CAY10585 caused no significant change in the cell count of vimentin+ cells; however, there was a profound reduction in the number of vimentin− cells. To ensure that the effect is target-related, an siRNA targeting HIF1α was tested, together with a scrambled siRNA/negative-control. Relative to the scrambled siRNA treatment, the HIF1α siRNA caused a small increase in the number of vimentin+ cells; however, there was a much larger reduction in the number of vimentin− cells, in concordance with the inhibitor data. The transfection of siRNA into MDA-MB-468 cells was performed as a reverse transfection. Lipofectamine 2000 (Life technologies, 0.25 uL per well) and siRNA (final concentration 40 nM) were separately diluted into 25 uL of Opti-MEM (Life technologies) and incubated for 5 minutes. The solutions were then combined and incubated for 15 minutes at room temperature. To this mixture, 8,000 cells diluted into 100 uL of DMEM with 10% FBS, and the combined mixture was seeded in a 96 well assay plate (Corning, #3603). To other wells, 8,000 MDA-MB-468 cells were seeded without any siRNA or transfection reagent. The plates were incubated over night at 37C/5% CO2. The next day, media in the transfected wells were replaced with fresh media. HIF1a inhibitor (CAY10585, sc-205346, Santa Cruz biotechnology) was diluted in DMSO (0.5% final concentration) and added to the untransfected cells at the concentrations indicated in the figure. As a control, cells were treated with 0.5% DMSO. All siRNA and inhibitor reactions were performed in triplicate. The cells were incu [file 12964_2015_106_MOESM7_ESM.png]
